# Registry Cohort Study to Determine Risk for Multiple Sclerosis after Vaccination for Pandemic Influenza A(H1N1) with Arepanrix, Manitoba, Canada

**DOI:** 10.3201/eid2407.161783

**Published:** 2018-07

**Authors:** Salaheddin M. Mahmud, Songul Bozat-Emre, Luiz C. Mostaço-Guidolin, Ruth Ann Marrie

**Affiliations:** University of Manitoba, Winnipeg, Manitoba, Canada (S.M. Mahmud, S. Bozat-Emre, R.A. Marrie);; Government of Manitoba, Winnipeg (L.C. Mostaço-Guidolin);; Health Sciences Centre, Winnipeg (R.A. Marrie)

**Keywords:** Multiple sclerosis, cohort studies, pandemic influenza vaccine, Adjuvant, viruses, Canada, vaccination, vaccines, influenza, Arepanix, risk

## Abstract

To investigate a potential risk for multiple sclerosis (MS) after vaccination with Arepanrix, the GlaxoSmithKline AS03-adjuvanted influenza A(H1N1)pdm09 vaccine, we used the provincewide immunization registry for Manitoba, Canada, to match 341,347 persons vaccinated during the 2009 pandemic to 485,941 unvaccinated persons on age, sex, address, and a propensity score measuring the probability of vaccination. We used a previously validated algorithm to identify MS cases from provincial hospital, physician, and prescription drug claims databases. After 12 months of follow-up, the age-adjusted incidence rate of MS was 17.7 cases per 100,000 person-years in the Arepanrix cohort and 24.2 per 100,000 in the unvaccinated cohort. The corresponding adjusted hazard ratio was 0.9. We observed similar patterns when we measured incidence over the entire follow-up period. The AS03 adjuvant, a candidate for inclusion in future pandemic vaccines, does not appear to increase the short-term risk for MS when included in influenza vaccines.

Multiple sclerosis (MS) is a chronic debilitating disease of the central nervous system (CNS) that affects >2.5 million persons worldwide (*1*). Its etiology is unknown but most likely is due to complex interactions between genetic and environmental factors (*2*). A role for infectious agents and vaccines has been suggested (*2*), but concrete evidence is lacking (*3*–*6*).

Soon after the 2009 influenza A(H1N1) pandemic, a signal of increased incidence of MS was detected in a postlicensure record-linkage study among residents of 3 counties in Sweden who received Pandemrix (GlaxoSmithKline, Dresden, Germany), an inactivated monovalent AS03-adjuvanted influenza A(H1N1)pdm09 vaccine (*7*). Another study from Stockholm, Sweden, reported increased risk for paraesthesias, but not of MS, among persons vaccinated with Pandemrix (*8*). Neither study was designed to assess an association with MS, and neither used validated algorithms for identifying MS from administrative databases. Because AS03, an adjuvant system containing α-tocopherol and squalene in an oil-in-water emulsion (*9*), is likely to be used in future pandemic vaccines, the European Medicine Agency mandated a study to evaluate the relationship between use of the GlaxoSmithKline AS03-adjuvanted pandemic vaccines and MS.

We assessed whether use of another AS03-adjuvanted A(H1N1)pdm09 vaccine, Arepanrix (GlaxoSmithKline, Quebec City, QC, Canada), was associated with increased risk for incident MS in Manitoba, Canada. Because of a combination of genetic and environmental factors, the prevalence of MS varies geographically (*2*). Canada is a high-prevalence region for MS. Within Canada, the prevalence of MS is particularly high in central and western provinces, such as Manitoba (*10*,*11*). Our secondary objective was to assess whether administration of Arepanrix was associated with increased risk for CNS demyelinating events that do not ultimately lead to MS (hereafter other demyelinating conditions).

## Methods

### Design and Data Sources

Manitoba Health is a government agency that provides publicly funded universal healthcare to virtually all of Manitoba’s 1.3 million residents. Insured services include hospital, physician, and preventive services, including vaccinations. All provided services are recorded in centralized electronic databases that can be linked using a unique lifetime personal health identification number (PHIN). A population registry tracks addresses and dates of birth, insurance coverage, and death for all insured persons. We analyzed population-based cohorts assembled by linking Manitoba Health’s vaccine registry with hospital, physician, and prescription claim databases, all part of a comprehensive repository of administrative and clinical databases housed at the Manitoba Centre for Health Policy (*12*). The Manitoba Immunization Monitoring System (MIMS) is a population-based provincewide registry of virtually all vaccines administered to Manitoba residents since 1988 (*13*). Vaccine type and date of vaccination are captured through direct data entry for vaccines administered by public health staff (who administered most influenza vaccines during the pandemic) or using physician claims data for vaccines administered by physicians (*13*).

Since 1971, the Hospital Abstracts Database recorded all hospital admissions in the province, including diagnoses and treatments coded using the International Classification of Diseases (ICD), Tenth Revision, and the Canadian Classification of Health Interventions (*12*). The Medical Services Database, also in operation since 1971, captures physician services including tariff codes for each service provided and a single ICD, Ninth Revision, diagnosis (*12*). The provincial Drug Program Information Network captures all out-of-hospital prescriptions dispensed in Manitoba since 1995 (*14*).

The study was approved by the Research Ethics Board of the University of Manitoba and the governmental Health Information Privacy Committee and registered with ClinicalTrials.gov (NCT02367222). Because this study was an European Medicine Agency regulatory requirement, patients were not involved in the development of research questions, study design, or conduct.

### Study Population

Anyone >6 months of age who was registered with Manitoba Health during September 15, 2009–March 15, 2010, when virtually all pandemic vaccines were administered, was eligible for inclusion in the study. We excluded participants who had <1 year of insurance coverage before enrollment (insufficient historical data) or >1 physician or hospitalization records for any demyelinating condition before enrollment.

### Determination of Vaccination Status

We obtained information about the receipt of the pandemic influenza, seasonal influenza, and other vaccines during and before the 2009–10 season from MIMS. Manitoba’s routine vaccination schedule includes seasonal trivalent inactivated influenza vaccines (TIVs); during the study period, vaccines used were were Fluviral (GlaxoSmithKline) and Vaxigrip (Sanofi Pasteur, Lyon, France). Most pandemic vaccines were administered during a mass immunization campaign that began October 26, 2009 (*15*). Like elsewhere in Canada, Arepanrix was used to vaccinate adults and children >6 months of age. Later, 2 unadjuvanted vaccines, from GlaxoSmithKline and CSL Limited (Parkville, VIC, Australia), were offered to pregnant women and children >10 years of age. As recommended by the World Health Organization, all vaccines contained 3.75 μg (per 0.5 mL) of hemagglutinin from an A/California/7/2009 (H1N1)v–like strain (X-179A). Because of limited supplies at campaign start, healthcare workers, Aboriginal persons, pregnant women, children 6–60 months of age, persons <65 years with chronic medical conditions, and all immunocompromised persons were prioritized (*15*).

To assemble study cohorts, we used a high-dimensional propensity score (PS) algorithm to calculate a PS for each eligible participant (*16*). PS is the conditional probability of receiving an intervention, an influenza A(H1N1)pdm09 vaccine in this case, given the value of a set of confounders (*17*). Use of PS in observational studies enables forming more comparable study groups by limiting comparisons to persons who had the same probability of receiving the intervention (*17*). This approach is particularly suitable for postlicensure studies of drug and vaccine safety in which the outcomes are rare, limiting the utility of conventional multivariable adjustment methods, but the intervention and confounder data are rich. The availability of vaccination status for the whole population (from MIMS) facilitated development and testing of the score.

We computed PS as the probability of receiving an influenza A(H1N1)pdm09 vaccine predicted by a logistic regression model that included vaccine receipt as the dependent variable and >400 independent variables including demographic information (e.g., socioeconomic status), coexisting illnesses, healthcare use (e.g., hospitalizations or physician visits), prescription drug use, and prior vaccinations. We matched each vaccinated person with a randomly selected unvaccinated person with the closest PS and the same age, sex, and neighborhood of residence.

### Study Endpoints

The primary endpoint was incidence of MS within 12 months after the index date. We defined the index date as the date of vaccination for vaccinated persons or the date of vaccination of the matched vaccinated person for unvaccinated persons. Secondary endpoints were incidence of MS until the end of follow-up (December 31, 2012) and of other demyelinating conditions within 12 months after the index date. We identified all endpoints by linking with Manitoba Health’s hospital, physician, and prescription claims databases. We used a previously validated algorithm, based on chart reviews, as well as separate medical record reviews and self-administered questionnaires, to identify cases (*10*). A case of MS was defined as >3 hospital, physician, or prescription claims for MS by an individual person. In validation studies in Manitoba and Nova Scotia (Canada), this definition had a positive predictive value of 80%–93% and a negative predictive value of 98% (*18*). The date of MS diagnosis was the date of the first medical contact for MS. Other demyelinating conditions were defined by >1 hospitalizations or >2 physician claims >30 days apart for any of the following: optic neuritis, acute transverse myelitis, demyelinating disease of CNS unspecified, other acute disseminated demyelination, or neuromyelitis optica (provided there was no subsequent MS diagnosis). We considered a case incident if no previous physician or hospitalization records indicated a diagnosis of any demyelinating condition going back to 1971.

### Covariates

Based on their postal codes, we assigned participants to a neighborhood of residence (neighborhood clusters within the capital city of Winnipeg and health districts in the rest of the province). We linked postal codes to 2006 Canadian census data to determine household income (quintiles) measured at the level of Census Dissemination areas. We used previously validated algorithms, based on the frequency of certain ICD codes, to identify various chronic diseases and health conditions including pregnancy (*19*–*21*).

### Statistical Analysis

For each endpoint, we calculated crude and age-standardized incidence rates (ASRs) and ratios (ARRs). We conducted survival analyses, measuring time-to-onset from the index date to the diagnosis date. Persons were censored on the earliest of study end date, loss to follow-up (because of death or immigration), or subsequent receipt of a different vaccine (because cases identified afterward might have resulted from the more recent vaccine). Two influenza vaccines given on the same day, typically an A(H1N1)pdm09 vaccine and a 2009–10 TIV, were considered as 1 episode. However, in analyses stratified by vaccine type, we grouped these episodes separately as the “concurrent A(H1N1)pdm09 vaccine/TIV” cohort and compared the incidence of MS in this group with that among persons who received only 1 vaccine: the “A(H1N1)pdm09 vaccine alone” cohort or the “TIV alone” cohort.

We estimated hazards ratios and corresponding 95% CIs associated with the receipt of an A(H1N1)pdm09 vaccine using Cox proportional hazard models with stratification on the matched pairs (to account for matching) (*22*). We verified the proportional hazards assumption using graphical and formal methods (*23*). We looked for effect modification with the receipt of the 2008–09 TIV, testing for interactions between A(H1N1)pdm09 vaccine and TIV terms using a likelihood ratio test with a liberal threshold for statistical significance (p<0.15). We also completed exploratory analyses to examine the association between unadjuvanted A(H1N1)pdm09 vaccines and MS. We could not adequately complete a planned subgroup analysis by age group and history of autoimmune diseases because of small MS numbers in most subgroups. Based on 341,000 vaccinated persons and MS incidence rate of 23 cases/100,000 persons among 485,000 nonvaccinated persons, our analysis had 95% power to detect a 20% increase in risk and 75% power to detect a 10% increase in risk, assuming a 2-sided α = 0.05 (*24*).

## Results

A total of 341,347 (29%) persons received >1 doses of an A(H1N1)pdm09 vaccine during the enrollment period. Of these, 278,131 (57%) received an A(H1N1)pdm09 vaccine only, 144,594 (30%) received a TIV only, and 63,216 (13%) received both. Almost all (96%) persons who received an A(H1N1)pdm09 vaccine received the adjuvanted Arepanrix either alone (78%) or in addition to a TIV (18%).

Although subcohorts might appear different when their aggregate characteristics are compared (Table 1), we based the actual analysis on the matched pairs (of vaccinated and unvaccinated matches) who were generally similar. As expected, children and younger adults dominated (54%) the A(H1N1)pdm09 vaccine group, whereas older (>55) adults (78%) and persons with chronic illnesses (30%) dominated the TIV group. There were more pregnant women in the vaccinated group, representing >50% of those who received the unadjuvanted A(H1N1)pdm09 vaccine. Vaccinated persons were more likely to have previously received the 2008–09 TIV and >1 pneumococcal vaccines.

**Table 1:** Cohort characteristics by vaccination status, Manitoba, Canada, 2009–2012*

Variable	No. (%) persons						
	Adjuvanted A(H1N1)pdm09			Unadjuvanted A(H1N1)pdm09		TIV alone	Unvaccinated
	Alone	And TIV		Alone	And TIV		
Total	267,539 (100)	61,239 (100)		10,592 (100)	1,977 (100)	144,594 (100)	485,941 (100)
Age group, y							
<14	83,097 (31.1)	10,206 (16.7)		1,245 (11.8)	109 (5.5)	4,915 (3.4)	99,463 (20.5)
15–34	62,261 (23.3)	12,637 (20.6)		4,717 (44.5)	679 (34.3)	7,094 (4.9)	92,008 (18.9)
35–44	38,401 (14.4)	9,272 (15.1)		1,835 (17.3)	363 (18.4)	6,463 (4.5)	51,795 (10.7)
45–54	39,958 (14.9)	12,018 (19.6)		1,452 (13.7)	389 (19.7)	12,696 (8.8)	74,454 (15.3)
>55	43,822 (16.4)	17,106 (27.9)		1,343 (12.7)	437 (22.1)	113,426 (78.4)	168,221 (34.6)
Sex							
F	144,461 (54.0)	31,081 (50.8)		7,352 (69.4)	1,194 (60.4)	82,868 (57.3)	266,956 (54.9)
Urban residence	146,256 (54.7)	41,916 (68.4)		6,202 (58.6)	1,604 (81.1)	96,605 (66.8)	292,583 (60.2)
Income quintile							
Q1 (lowest)	53,269 (19.9)	9,766 (15.9)		1,803 (17.0)	279 (14.1)	27,899 (19.3)	92,147 (19.0)
Q2	47,036 (17.6)	11,066 (18.1)		1,833 (17.3)	355 (18.0)	27,593 (19.1)	91,214 (18.8)
Q3	48,257 (18.0)	10,976 (17.9)		1,766 (16.7)	358 (18.1)	28,037 (19.4)	91,542 (18.8)
Q4	50,592 (18.9)	12,454 (20.3)		2,373 (22.4)	447 (22.6)	27,167 (18.8)	95,379 (19.6)
Q5 (highest)	62,320 (23.3)	15,602 (25.5)		2,613 (24.7)	503 (25.4)	25,103 (17.4)	101,411 (20.9)
Cannot be calculated	6,065 (2.3)	1,375 (2.2)		204 (1.9)	35 (1.8)	8,795 (6.1)	14,248 (2.9)
Immunosuppressed	11,541 (4.3)	4,028 (6.6)		322 (3.0)	78 (3.9)	20,990 (14.5)	33,561 (6.9)
Autoimmune diseases	6,680 (2.5)	2,669 (4.4)		197 (1.9)	45 (2.3)	10,179 (7.0)	17,661 (3.6)
Any chronic diseases	18486 (6.9)	7,485 (12.2)		364 (3.4)	96 (4.9)	42,909 (29.7)	65,158 (13.4)
Pregnant, % of all 15–49-year-old females	2,184 (3.1)	355 (2.4)		2,857 (50.6)	330 (40.4)	816 (7.4)	5,160 (4.9)
High priority for A(H1N1)pdm09 vaccine	142,909 (53.4)	25,652 (41.9)		6,200 (58.5)	683 (34.5)	59,165 (40.9)	230,948 (47.5)
High priority for TIV	42,207 (15.8)	12,958 (21.2)		3,231 (30.5)	419 (21.2)	105,557 (73.0)	158,565 (32.6)
Received 2008–09 TIV	41,896 (15.7)	22,231 (36.3)		1,237 (11.7)	542 (27.4)	109,107 (75.5)	42,071 (8.7)
Received pneumococcal vaccine	49,203 (18.4)	11,554 (18.9)		286 (2.7)	64 (3.2)	87,552 (60.6)	86,261 (17.8)

*A(H1N1)pdm09, pandemic influenza A(H1N1) strain; Q, quintile; TIV, trivalent influenza vaccine.

By the end of the first year of follow-up, a total of 106 incident MS cases had been diagnosed among the unvaccinated cohort (Table 2), corresponding to an ASR of 24.2 (95% CI 20.1–28.3)/100,000 person-years, compared with 69 cases and an ASR of 20.2 (95% CI 15.4–24.9)/100,000 person-years among persons who received any influenza vaccine. The ASR was lower for persons who received Arepanrix (17.7 [95% CI 14.1–21.2]/100,000 person-years), corresponding to an ARR of 0.7 [95% CI 0.3–1.7]. The rate was similar for the unadjuvanted vaccine cohort. The ASR was higher for persons who received the 2009–10 TIV alone (36.8 [95% CI 25.0–48.6]/100,000 person-years) than for those who did not (ARR 1.5 [95% CI 0.3–6.8]). We observed no increase in risk for persons who received the TIV and A(H1N1)pdm09 vaccine concurrently. Regardless of vaccine type, ARRs calculated over the entire period (median of 3 years) were consistent with lack of association with vaccine administration.

**Table 2:** Crude and age-standardized rates of incident multiple sclerosis and influenza vaccination status, Manitoba, Canada, 2009–2012*

Vaccination status	No. events	Rate (95% CI)			Rate ratio (95% CI)	
Crude	Age-standardized	Crude	Age-adjusted
1 year after index date						
Unvaccinated	106	23.2 (19.2–28.0)	24.2 (20.1–28.3)		1	1
Vaccinated A(H1N1) pdm09/TIV	69	19.1 (15.1–24.2)	20.2 (15.4–24.9)		0.8 (0.6–1.1)	0.8 (0.3–2.2)
A(H1N1)pdm09 alone	43	17.6 (13.1–23.8)	17.7 (14.1–21.2)		0.8 (0.5–1.1)	0.7 (0.3–1.7)
Concurrent A(H1N1)pdm09/TIV	12	20.3 (11.5–35.7)	19.4 (8.6–30.2)		0.9 (0.5–1.6)	0.8 (0.1–5.0)
TIV alone	14	24.3 (14.4–41.1)	36.8 (25.0–48.6)		1.0 (0.6–1.8)	1.5 (0.3–6.8)
Adjuvanted A(H1N1)pdm09 alone	40	17.1 (12.5–23.3)	17.4 (13.8–21.1)		0.7 (0.5–1.1)	0.7 (0.3–1.7)
Concurrent adjuvanted A(H1N1)pdm09/TIV	11	19.2 (10.6–34.7)	18.3 (7.3–29.3)		0.8 (0.4–1.5)	0.8 (0.1–5.1)
Unadjuvanted A(H1N1)pdm09 alone	s	s	18.6 (8.8–28.3)		1.3 (0.4–4.2)	0.8 (0.1–4.2)
Concurrent unadjuvanted A(H1N1)pdm09/TIV	s	s	37.9 (13.8–62.0)		2.3 (0.3–16.4)	1.6 (0.1–26.6)
Entire follow-up period						
Unvaccinated	188	15.6 (13.5–18.0)	16.0 (13.5–18.5)		1	1
Vaccinated A(H1N1)pdm09/TIV	132	15.1 (12.7–17.9)	15.4 (12.4–18.4)		1.0 (0.8–1.2)	1.0 (0.5–1.9)
A(H1N1)pdm09 alone	82	14.6 (11.7–18.1)	14.9 (9.3–20.5)		0.9 (0.7–1.2)	0.9 (0.3–2.8)
Concurrent A(H1N1)pdm09/TIV	33	20.1 (14.3–28.2)	18.2 (11.8–24.6)		1.3 (0.9–1.9)	1.1 (0.4–3.6)
TIV alone	17	11.4 (7.1–18.4)	16.6 (9.5–23.8)		0.7 (0.4–1.2)	1.0 (0.3–3.9)
Adjuvanted A(H1N1)pdm09 alone	78	14.5 (11.6–18.0)	15.0 (9.3–20.7)		0.9 (0.7–1.2)	0.9 (0.3–2.9)
Concurrent adjuvanted A(H1N1)pdm09/TIV	32	20.1 (14.2–28.4)	18.4 (11.9–24.9)		1.3 (0.9–1.9)	1.1 (0.4–3.7)
Unadjuvanted A(H1N1)pdm09 alone	s	s	9.7 (3.6–15.8)		1.1 (0.4–2.9)	0.6 (0.1–2.6)
Unadjuvanted A(H1N1)pdm09/TIV	s	s	13.1 (0.0–27.2)		1.2 (0.2–8.5)	0.8 (0.0–13.5)

*Rates are per 100,000 person-years. A(H1N1)pdm09, pandemic influenza A(H1N1) strain; s, suppressed because of small sample size (n = 1–5) in accordance with the requirements of the data custodian; TIV, trivalent influenza vaccine.

After 1 year of follow-up, only 27 persons among the unvaccinated cohort met the case definition for having other demyelinating conditions, corresponding to an ASR of 6.9 (95% CI 2.6–11.1)/100,000 person-years, compared with 17 cases and an ASR of 4.7 (95% CI 0.0–10.6)/100,000 person-years among the vaccinated cohort (Table 3). Generally, ARRs calculated over this period were consistent with lack of an association with vaccine administration. Findings were similar over the longer follow-up period, except for persons who received an unadjuvanted vaccine where the ASR was higher (7.4 [95% CI 0.0–18.0]/100,000 person-years), but because of the small number of cases (<6), the corresponding ARR (2.1) was imprecise (95% CI 0.1–39.9).

**Table 3:** Crude and age-standardized rates of incident demyelinating conditions not ultimately diagnosed as multiple sclerosis and influenza vaccination status, Manitoba, Canada, 2009–2012*

Vaccination status	No. events	Rate (95% CI)			Rate ratio (95% CI)	
Crude	Age-standardized	Crude	Age-adjusted
1 year after index date						
Unvaccinated	27	5.9 (4.1–8.6)	6.9 (2.6–11.1)		1	1
Vaccinated A(H1N1)pdm09/TIV	17	4.7 (2.9–7.6)	4.7 (0.0–10.6)		0.8 (0.4–1.5)	0.7 (0.1–6.4)
A(H1N1) pdm09 alone	s	s	5.6 (0.0–13.3)		0.8 (0.4–1.6)	0.8 (0.1–10.6)
Concurrent A(H1N1) pdm09/TIV	s	s	7.8 (0.0–17.0)		1.4 (0.6–3.7)	1.1 (0.1–15.2)
TIV alone	0	0	NA		NA	NA
Adjuvanted A(H1N1)pdm09 alone	11	4.7 (2.6–8.5)	5.4 (0.0–13.1)		0.8 (0.4–1.6)	0.8 (0.1–10.8)
Concurrent adjuvanted A(H1N1)pdm09/TIV	s	s	8.0 (0.0–17.3)		1.5 (0.6–3.8)	1.2 (0.1–15.5)
Unadjuvanted A(H1N1)pdm09 alone	s	s	6.2 (0.0–16.0)		1.7 (0.2–12.8)	0.9 (0.0–18.1)
Unadjuvanted A(H1N1)pdm09/TIV	0	0	NA		NA	NA
Entire follow-up period						
Unvaccinated	40	3.3 (2.4–4.5)	3.6 (0.8–6.3)		1	1
Vaccinated A(H1N1)pdm09/TIV	25	2.9 (1.9–4.2)	2.9 (0.0–7.1)		0.9 (0.5–1.4)	0.8 (0.1–6.2)
A(H1N1)pdm09 alone	17	3.0 (1.9–4.9)	3.5 (0.0–9.1)		0.9 (0.5–1.6)	1.0 (0.1–10.3)
Concurrent A(H1N1)pdm09/TIV	s	s	3.8 (0.0–10.7)		1.1 (0.5–2.6)	1.1 (0.1–15.6)
TIV alone	s	s	0.4 (0.0–2.1)		0.4 (0.1–1.7)	0.1 (0.0–1.0)
Adjuvanted A(H1N1)pdm09 alone	15	2.8 (1.7–4.6)	3.2 (0.0–8.9)		0.8 (0.5–1.5)	0.9 (0.1–10.5)
Concurrent adjuvanted A(H1N1)pdm09/TIV	6	3.8 (1.7–8.4)	4.0 (0.0–10.9)		1.1 (0.5–2.7)	1.1 (0.1–16.1)
Unadjuvanted A(H1N1) alone	s	s	7.4 (0.0–18.0)		2.5 (0.6–10.5)	2.1 (0.1–39.9)
Unadjuvanted A(H1N1)pdm09/TIV	0	0	NA		NA	NA

*Rates are per 100,000 person-years. A(H1N1)pdm09, pandemic influenza A(H1N1) strain; NA, cannot be estimated; s, suppressed because of small sample size (n = 1–5) in accordance with the requirements of the data custodian; TIV, trivalent influenza vaccine.

In Cox models adjusted for matching (model A), we found no evidence of an association between MS and the receipt of any vaccine (Table 4). For instance, the hazard ratio for receipt of Arepanrix alone was 0.9 (95% CI 0.6–1.4) and did not appreciably change with further adjustment for receipt of the 2008–09 TIV (model B). We observed similar patterns when we measured disease occurrence over the entire follow-up period (Table 4).

**Table 4:** Association between influenza vaccination and incidence of multiple sclerosis and demyelinating conditions not ultimately diagnosed as multiple sclerosis, Manitoba, Canada, 2009–2012*

Vaccination status	Multiple sclerosis, HR (95% CI)			Other demyelinating conditions, HR (95% CI)	
Model A	Model B		Model A	Model B
1 Year after index date					
Unvaccinated	Ref	Ref		Ref	Ref
Vaccinated A(H1N1)pdm09/TIV	0.9 (0.6–1.2)	0.9 (0.6–1.3)		0.6 (0.3–1.2)	0.6 (0.3–1.2)
A(H1N1) pdm09 alone	0.9 (0.6–1.3)	0.9 (0.6–1.4)		0.5 (0.3–1.1)	0.5 (0.3–1.2)
Concurrent A(H1N1) pdm09/TIV	0.6 (0.3–1.3)	0.6 (0.3–1.4)		2.0 (0.4–10.9)	2.3 (0.4–15)
TIV alone	1.1 (0.5–2.3)	1.2 (0.5–2.9)		NA	NA
Adjuvanted A(H1N1)pdm09 alone	0.9 (0.6–1.4)	0.9 (0.6–1.5)		0.5 (0.2–1.1)	0.5 (0.2–1.1)
Concurrent adjuvanted A(H1N1)pdm09/TIV	0.6 (0.3–1.3)	0.6 (0.3–1.3)		2.0 (0.4–10.9)	2.3 (0.4–14.8)
Unadjuvanted A(H1N1)pdm09 alone	0.8 (0.2–3.4)	0.8 (0.2–3.5)		1.0 (0.1–15.9)	1.0 (0.1–15.9)
Unadjuvanted A(H1N1)pdm09/TIV	1.0 (0.1–15.9)	1.1 (0.1–18.0)		NA	NA
Entire follow-up period					
Unvaccinated	Ref	Ref		Ref	Ref
Vaccinated A(H1N1)pdm09/TIV	1.0 (0.8–1.3)	1.1 (0.8–1.5)		0.7 (0.4–1.1)	0.7 (0.4–1.2)
A(H1N1) pdm09 alone	1.0 (0.8–1.4)	1.1 (0.8–1.5)		0.5 (0.3–1.0)	0.6 (0.3–1.1)
Concurrent A(H1N1) pdm09/TIV	1.1 (0.6–1.8)	1.2 (0.7–2)		1.7 (0.4–7)	2.1 (0.4–10.2)
TIV alone	0.9 (0.5–1.9)	1.2 (0.6–2.5)		1.0 (0.1–7.1)	1.5 (0.1–16.1)
Adjuvanted A(H1N1)pdm09 alone	1.0 (0.8–1.4)	1.1 (0.8–1.5)		0.5 (0.3–1.0)	0.5 (0.3–1.1)
Concurrent adjuvanted A(H1N1)pdm09/TIV	1.1 (0.6–1.8)	1.2 (0.7–2.0)		1.7 (0.4–7.0)	2.1 (0.4–10.1)
Unadjuvanted A(H1N1)pdm09 alone	0.8 (0.2–3.0)	0.8 (0.2–3.1)		1.0 (0.1–15.9)	1.0 (0.1–15.9)
Unadjuvanted A(H1N1)pdm09/TIV	1.0 (0.1–15.9)	1.2 (0.1–19.8)		NA	NA

*Model A estimates were adjusted for matching variables (propensity scores, age, sex, and area of residence). Model B estimates also were adjusted for receipt of the 2008–09 TIV. A(H1N1)pdm09, pandemic influenza A(H1N1) strain; HR, hazard ratio; NA, cannot be estimated; ref, referent; TIV, trivalent influenza vaccine; TIV.

In Cox models, we found no evidence of increased risk for other demyelinating conditions with the receipt of a pandemic vaccine (Table 4). The receipt of either TIV alone or concurrently with the adjuvanted pandemic vaccine was associated with an increased risk for these conditions and the association persisted after adjusting for receipt of the 2008–09 TIV and when repeated for the entire study period. However, although these findings were consistent, none of these associations was statistically significant or precise because few cases were diagnosed among these groups.

## Discussion

In this large population-based registry study, we found no evidence of an association between the adjuvanted influenza A(H1N1)pdm09 vaccine used in Canada and the incidence of MS or that of other acquired CNS demyelinating disorders. These findings are largely consistent with limited prior work regarding the A(H1N1)pdm09 vaccine and other influenza vaccines.

Few studies have examined the association between the A(H1N1)pdm09 vaccine and occurrence of MS. In published (mostly manufacturer-sponsored) randomized controlled trials conducted during the pandemic, there were no reports of clinically significant adverse events (including MS) of the different pandemic vaccine formulations (*25*). These findings are reassuring, but these trials might have not been large enough to detect a small increase in risk.

Similarly, vaccine adverse events surveillance systems in Europe and the United States did not detect increased risk for MS with pandemic vaccine use (*26*,*27*). No increased risk was found in an analysis of the European EudraVigilance database, which tracked reports of suspected autoimmune disorders after use of either adjuvanted (including Pandemrix) or unadjuvanted A(H1N1)pdm09 vaccines (*26*). Analyses of the US Vaccine Adverse Event Reporting System reached a similar conclusion (*27*).

A large retrospective record-linkage study from Sweden reported increased risk for paresthesias, but not of MS, among persons vaccinated with Pandemrix (*8*,*28*). Among persons with a high risk for influenza complications who were mostly vaccinated in the first 45 days of the campaign (healthcare workers, children, pregnant women, and persons with chronic diseases), the risk for MS was 1.17 (95% CI 0.82–1.66), and the risk estimates were highest within 6 weeks after vaccination (1.35 [95% CI 0.68–2.67]). There was no similar increase of risk among other groups. The authors attributed the excess risk among high-risk groups to possible confounding by underlying co-morbidity and vaccine indication.

Other AS03-adjuvanted influenza vaccines (e.g., avian influenza [H5N1] vaccines), as well as vaccines based on other oil-in-water adjuvants (e.g., MF59), were found in several randomized controlled trials to be more reactogenic than unadjuvanted TIVs. However, no serious adverse events, including MS, were reported (*29*). Finally, no evidence exists to support a link with use of unadjuvanted TIVs. In a systematic review, the authors identified 4 observational studies; the pooled risk ratio of developing MS after influenza vaccination was 0.97 (95% CI 0.77–1.23) with little evidence of heterogeneity (p = 0.368) (*30*).

We did not evaluate the association between A(H1N1)pdm09 vaccines and relapses in persons with established MS. The evidence for this association has been inconsistent. In several small randomized controlled trials, there was no increased incidence of relapses after A(H1N1)pdm09 vaccination (*31*,*32*). Observational studies provided conflicting findings, probably because of limitations in study design, such as small sample sizes and selection biases (*33*,*34*). Earlier studies and systematic reviews have not found any evidence of an increased risk for relapse with use of TIVs and other vaccines (*35*,*36*).

Evidence is scarce for an association between A(H1N1)pdm09 vaccination and other CNS demyelinating disorders. In a review of published case series, postmarketing surveillance data, and observational studies, a diagnosis of optic neuritis was not associated with influenza vaccination (*37*). Although 13 cases were reported after influenza vaccination, no association was found in 2 case–control studies (*37*). A more recent case–control study did not find an association between vaccine use in general and subsequent CNS demyelinating disorders (*38*).

We had access to accurate records of hospitalization, physician utilization, vaccination, and prescriptions for the entire population, which reduced the possibilities of selection bias and differential misclassification of exposures and outcomes and ensured that unvaccinated persons were truly enrolled and their healthcare use was captured. However, some variables might have been measured with error. The ascertainment of MS cases is most likely incomplete because initial symptoms might not be recognized as demyelinating, and diagnostic delays occur, although these have declined substantially over time in Manitoba (*10*) and elsewhere, such that most MS is diagnosed <1 year from onset (*39*). However, the incidence rate of MS for unvaccinated persons in this study was comparable with rates measured in similarly young populations in earlier studies from Manitoba and elsewhere (*10*,*40*). Furthermore, underascertainment is probably nondifferential with respect to A(H1N1)pdm09 vaccination because knowledge of vaccination status is unlikely to have directly influenced the way MS was diagnosed or coded, suggesting that our relative risk estimates of the association are likely to be accurate, even though our absolute MS incidence rates might be underestimated.

We did not have information about putative lifestyle and environmental risk factors. We attempted to adjust for these factors by matching on age, sex, region of residence, and PS. Matching on region reduces the likelihood of confounding by ethnicity because ethnic minorities tend to cluster in communities, even in the province’s large urban centers. PS reduces confounding by measured (e.g., access to healthcare) and unmeasured (e.g., smoking) confounders because of the inclusion of proxy conditions (e.g., smoking-related diseases) in the PS calculation. Furthermore, the vaccinated and unvaccinated cohorts were comparable, indicating a reasonable performance of the matching procedure. However, residual confounding remains possible.

Although the relatively large sample size permitted calculation of reasonably precise estimates, small numbers in some subgroup analyses limited the precision of estimates. Generalizability of the findings to other populations depends on their geographic location, ethnic composition, and access to the pandemic vaccine products. Manitoba’s population tends to be typical of many western populations, especially those in northern high-latitude countries, in terms of MS incidence and ethnic composition and even with the timing and epidemiology of the 2009 pandemic and the nature of the public health response to the pandemic. Finally, our findings might not be applicable to a future pandemic if the causative virus has a drastically different risk profile. However, we found no evidence that influenza virus antigenicity or pathogenicity influences the safety of commonly used adjuvant. Thus, our findings about the safety of the AS03 adjuvant are likely to hold true.

In conclusion, we found no evidence of increased short-term risk for MS or other acquired CNS demyelinating conditions after vaccination with an AS03-adjuvanted influenza A(H1N1)pdm09 vaccine. Therefore, it does not seem that the AS03 adjuvant, a candidate for inclusion in future pandemic vaccines, changes the safety profile of influenza vaccines with regard to MS. Although consistent with the lack of association with vaccination observed in the literature, our study findings do not rule out an increased (or decreased) risk for MS over the long term. Therefore, a similar analysis should be conducted once sufficient time has passed since the pandemic.

|||||||||||||||||||||||||||||||||||||||||||
